# Homeobox a5 Promotes White Adipose Tissue Browning Through Inhibition of the Tenascin C/Toll-Like Receptor 4/Nuclear Factor Kappa B Inflammatory Signaling in Mice

**DOI:** 10.3389/fimmu.2018.00647

**Published:** 2018-03-29

**Authors:** Weina Cao, Hongtao Huang, Tianyu Xia, Chenlong Liu, Saeed Muhammad, Chao Sun

**Affiliations:** College of Animal Science and Technology, Northwest A&F University, Yangling, China

**Keywords:** homeobox a5, adipocyte, BMP4, browning, inflammasome, methylation

## Abstract

Lipopolysaccharide (LPS) induces rapid increase in systemic inflammatory factors. As adipose tissue is a key contributor to the inflammatory response to numerous metabolic stimuli, it is important to understand the mechanism behind the LPS-induced inflammation in white adipose tissue (WAT). Homeobox a5 (Hoxa5) is an important transcription factor, which is highly expressed in adipose tissue, and its mRNA expression is increased at cold exposure in mice. So far, the function of Hoxa5 in adipose tissue browning has been poorly understood. So, the objective of this study was conducted to determine the role of Hoxa5 in adipose inflammatory response and white adipose browning in mice. LPS-induced inflammatory and cold-induced browning model were conducted. We compared the coordinated role of Hoxa5 in inflammation and thermogenesis of mice adipose. Transcriptional and methylation regulation was determined by luciferase assay, electrophoretic mobility shift assay, and bisulfite conversion experiment. Hoxa5 and tenascin C (TNC) were involved in WAT inflammation and browning in mice with LPS injection. Furthermore, Hoxa5 inhibited the TNC-involved activation of Toll-like receptor (TLR) 4/nuclear factor kappa B (NF-κB) signal pathway and promoted WAT browning. Moreover, we found that a BMP4/Smad1 signal, closely related to browning, was activated by Hoxa5. Hoxa5 relieved adipocyte inflammation by decreasing TNC-mediated TLR4 transducer and activator of the NF-κB pathway. Interestingly, descended methylation level increased Hoxa5 expression in cold exposure. Our findings demonstrated that Hoxa5 alleviated inflammation and enhanced browning of adipose tissue *via* negative control of TNC/TLR4/NF-κB inflammatory signaling and activating BMP4/Smad1 pathway. These findings indicated a novel potential means for the regulation of inflammation in adipocytes to prevent obesity and other inflammatory diseases.

## Introduction

Obesity has become a public health epidemic worldwide, and adipose tissues take an important part in the development of obesity (1, 2). Obesity-associated metabolic diseases and type II diabetes are accompanied by a condition of adipose tissue inflammation (3–5). Mammals contain two types of adipose tissue, white adipose tissue (WAT) and brown adipose tissue (BAT). WAT stores surplus energy, whereas BAT has a unique thermogenic capacity that can resist obesity and hypothermia (6–8). Recently, WAT is verified to transform into a “brown-like” state with cold stimulation or by β_3_-adrenergic receptor agonists (9, 10). The advance of iatreusiology to increase WAT browning might reflect a potential cure for obesity-related diseases (11).

The homeobox a5 (Hoxa5) gene, a developmental transcription factor, differentially expresses between adipose tissue depots (12). Hoxa5 expresses at a higher level in visceral adipose tissue and BAT than that in subcutaneous depots (13). The Hoxa5 expression is decreased with a high-fat diet in mice adipose tissue, while increased in human adipose tissue after fat loss surgery (14, 15). It is reported that Hox5 mutant mice have increased lung inflammation, and HOXA5 can be applied to vascular inflammation (16, 17). However, the regulatory mechanism of Hoxa5 on adipose inflammation and browning still needs to be illustrated.

In this issue, we studied the effects of Hoxa5 on lipopolysaccharide (LPS)-induced inflammatory response as well as WAT browning. We found that Hoxa5 promotes inflammation-reduced WAT browning by inhibiting tenascin C (TNC)-mediated Toll-like receptor (TLR) 4/nuclear factor kappa B (NF-κB) signal pathway. Moreover, Hoxa5 activated BMP4/Smad1 pathway leading to promotion of WAT browning. Thus, our findings indicated a novel mechanistic relevance among Hoxa5, inflammation, and WAT browning, and open new therapeutic possibilities against obesity and inflammation.

## Materials and Methods

### Animal Experiment

Four-week-old C57BL/6 male mice were used, and feeding procedure for mice was as described in our previous reports in detail (18). Purified products of overexpression Hoxa5 adenovirus vector (pAd-Hoxa5), adenovirus interference vector of Hoxa5 (sh-Hoxa5), or empty adenovirus vector (pAd-control) at the titer of 1 × 10^12^ IFU/mL were subcutaneously injected into 8-week-old mice for 10 days, respectively. LPS (1 mg/kg body weight) injection (19) was performed 12 h before slaughtered and cold exposure at 4°C for 4 h before slaughtered. Then the mice were euthanized for collection of inguinal adipose tissue and blood.

### Primary Adipocytes Culture and Virus Vectors Infection

Epididymal WATs from 4-week-old mice were harvested for cells. The adipocyte culture was performed as previously described (20). Cells were differentiated after induction for 4 days. The pre-adipocytes were infected with overexpression Hoxa5 adenovirus vector (pc-Hoxa5), adenovirus interference vector of Hoxa5 (sh-Hoxa5), and overexpression TNC adenovirus vector (pc-TNC) for 48 h at the titer of 1 × 10^9^ IFU/mL and then to induce differentiation.

### RNA-Seq Analysis

Inguinal white adipose tissue (iWAT) from 4-week-old mice was harvested for experimental cells. The cells were differentiated after induction for 4 days. The pre-adipocytes were infected with purified products of overexpression Hoxa5 adenovirus vector (pAd-Hoxa5) or empty adenovirus vector (control). The total RNA from the differentiated adipocytes had infected with an overexpression Hoxa5 adenovirus vector, or empty adenovirus vector was prepared with RNAiso Reagent (Takara, China, D312), and the RNA-seq analysis was performed as previously described (21). Briefly, quantification and quality control of the sample libraries were assessed by Agilent 2100 Bioanalyzer and ABI StepOnePlus Real-time PCR System. RNA sequencing was performed using Hiseq 4000 instrument (Illumina, San Diego, CA, USA). Real-time analysis was used for base calling. Fastq files were mapped to the mouse genome (NCBI37/mm9) using TopHat (version 2.0.4, Johns Hopkins University, Baltimore, MD, USA). Mapped reads were then assembled *via* Cufflinks (version 2.0.2, University of Washington Seattle, WA, USA) with the default settings. Assembled transcripts were then merged by using the Cuffmerge program with the reference genome. Analysis of mRNA levels was carried out using the Cuffdiff program, and samples were grouped for treatment condition, three replicates per group. Gene Ontology (GO), clustering analysis, and pathway enrichment analysis were performed to categorize the considerably enriched functional classification pathways in which DEGs operated.

### Drug Treatments

Cells were incubated with LPS (1 µg/mL, Sigma, St. Louis, MO, USA) for 6 h Adipocytes were treated with 1 µM ATRA (Sigma, St. Louis, MO, USA) for 24 h. The Cl316,243 (1 µM, Sigma, St. Louis, MO, USA) was mixed to treat adipocytes for 4 h. 5-Azacytidine (Sigma, St. Louis, MO, USA) working solution (5 µM) was prepared to treat cells after transfection. Inflammasome was activated by ATP (4 mM) for 60 min. Cells were treated with LDN193,189 (10 µM, Selleck, Houston, TX, USA) for 4 h to block BMP4/Smad1 signal pathway. TNC on TLR4 or NF-κB signal pathway was suppressed by TAK-242 (10 µM, MedChemExpress, Monmouth Junction, USA) for 6 h or BAY11-7082 (100 µM, Selleck, Houston, TX, USA) for 4 h, respectively.

### Immunohistochemistry and Immunofluorescence

The frozen sections of adipose were prepared, and antigen retrieval and Endogenous peroxidase activity blocking were performed. Anti-PGC1α (Abcam) and biotinylated secondary antibodies (1:3,000) were used for incubation.

The frozen sections of adipose or the cells were incubated in 4% paraformaldehyde for 20 min and then were permeabilized by 0.1% Triton X-100 for 10 min. Non-specific binding was blocked with 5% BSA for 30 min. Frozen sections or cells were incubated with primary antibody against NLRP3, IL1β, PGC1α, and NF-κB (Abcam, England) at 37°C for 2 h. Then frozen sections or cells were stained at a 1:100 dilution in 2% BSA of Cy3-conjugated (or FITC-conjugated) Donkey Anti-Rabbit IgG (Sangon Biotech, China) for 30 min. All of the washes were done in 1× PBS. An anti-fade solution containing DAPI (Solarbio, China) was used.

### Luciferase Report Assay

Fragments containing TNC promoter sequences were subcloned into a pGL3-basic vector (Takara, China). HEK293T or 3T3-L1 cells were cultured in 24-well plates and co-transfected with TNC promoter plasmid and pc-Hoxa5 plasmid or pcDNA3.1 plasmid. After 48 h, then cells were harvested to analyze the activity using the Dual-Luciferase Reporter assay system (Promega, USA). The details were performed as previous addressed (22).

### Electrophoretic Mobility Shift Assay (EMSA)

We prepared nuclear protein extracted from adipocytes as previously described (23). Experimental steps followed according to the Light Shift1 Chemiluminescent EMSA Kit (Pierce Corp., Rockford, IL, USA) manufacturer’ s instructions.

### DNA Methylation Assay

Bisulfite treatment of genomic DNA was performed with EZ DNA Methylation-Gold™ Kit (Zymo Research), followed the manufacturer’ s instructions. For bisulfite sequencing, PCR fragments were cloned with pGEMT-Easy vector system (Promega), and 10 clones were sequenced to determine the methylation status, and the experimental procedure was described in our previous reports in detail (24).

### Enzyme-Linked Immunosorbent Assay

The measurement of protein levels of TNFα and IL-6 in mouse serum was conducted using the commercial ELISA kits from Sigma-Aldrich (St. Louis, MO, USA) according to the manufacturer’ s instructions.

### Real-Time Quantitative PCR Analysis

Primers for Hoxa5, UCP1, PGC1α, IL1β, IL6, MCP1, TLR4, TNC, NLRP3, Dnmt1, Dnmt3a, and Mbd3 were synthesized by Shanghai Sangon Ltd. (Shanghai, China). Procedure for quantitative PCR was described in our previous reports in detail (25).

### Western Blot Analysis

Antibodies against UCP1 (ab10983), PGC1α (ab72230), PRDM16 (ab106410), IL1β (ab200478), IL6 (ab7737), TNFα (ab8348), TLR4 (ab13556), NLRP3 (ab210491), BMP4 (ab155033), p-Smad1 (ab73211), p65NF-κB (ab16502), IĸBα (ab32518), and GAPDH (ab9484) were from Abcam (Cambridge, England). The experimental procedure was described in our previous reports in detail (26).

### Statistical Analysis

Statistical analyses were conducted using SAS v8.0 (SAS Institute, Cary, NC, USA). Data were determined using the one-way ANOVA. Comparisons among individual means were made by Fisher’s least significant difference. Data are presented as mean ± SD; *p* < 0.05 was considered to be significant.

## Results

### Hoxa5 Downregulated Inflammation Genes and Upregulated Thermogenic Genes in Mice Adipocytes

**Figure 1:**
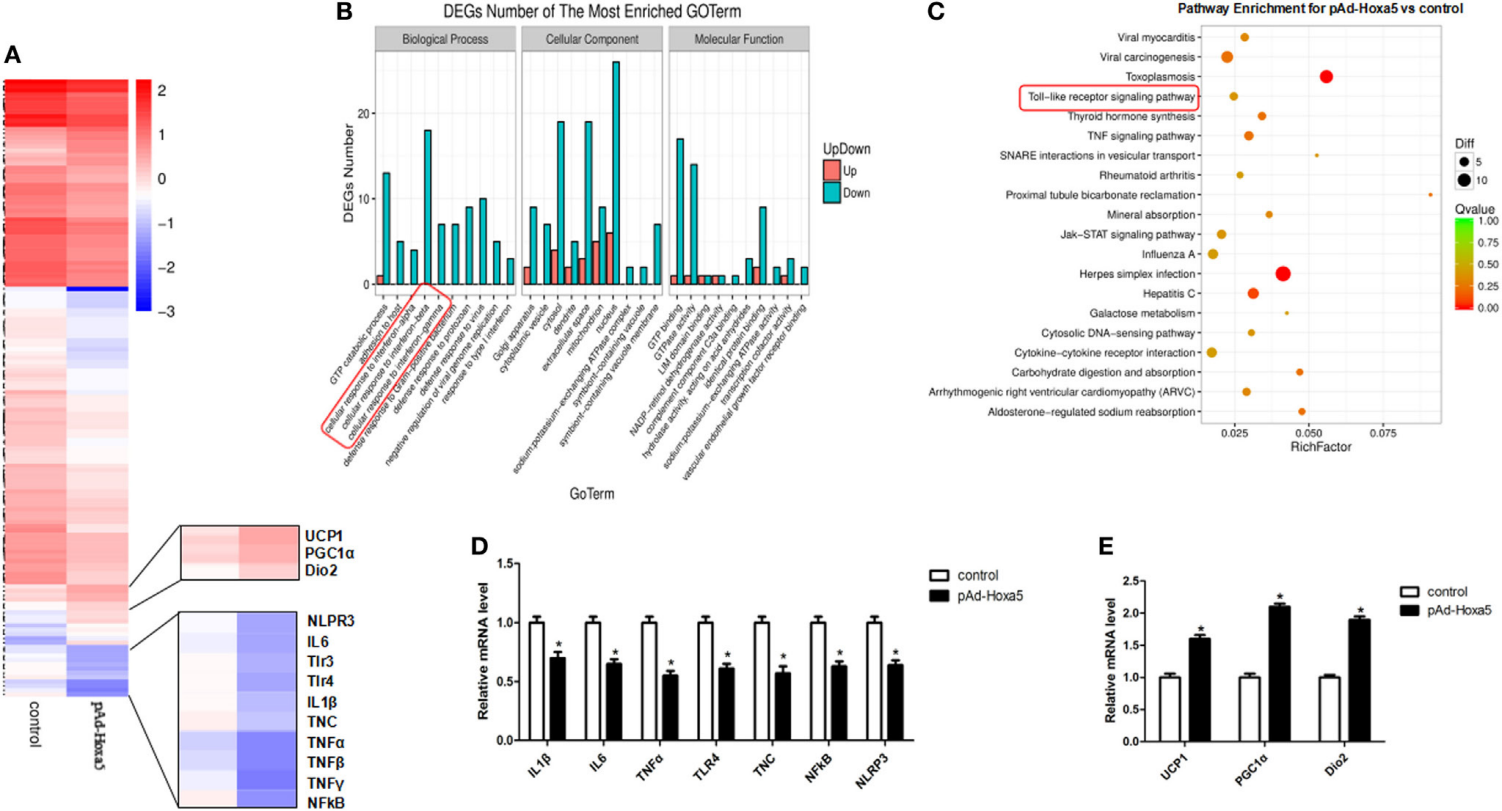
Overexpression homeobox a5 (Hoxa5) downregulated inflammation genes and upregulated thermogenic genes in adipocytes. **(A)** Heatmap of genes set from RNA-seq data with forced expression of Hoxa5 in mice adipocytes, along with the top affected genes (*n* = 3). **(B)** Gene ontology (GO) analysis of the altered genes in **(A)** (*n* = 3). **(C)** Analysis of altered pathway enrichment in response to forced expression of Hoxa5 (*n* = 3). **(D,E)** Changes in the mRNA level of genes associated with inflammation and browning, which were significantly altered from RNA-seq analysis (*n* = 3). Values are represented as means ± SEM. *p < 0.05 compared with the control group.

To explore the effect of Hoxa5 on adipose tissue, we used RNA-Seq to compare adipocytes transcriptomes treated with overexpression of Hoxa5 or control. Forced expression of Hoxa5 resulted in an anti-inflammatory with pro-thermogenesis transcriptional signature defined by the downregulation of 186 genes and upregulation of 39 genes when significant gene expression differences are grouped and visualized as a Heatmap (Figure 1A). Further analyses revealed that signature genes expressions altered by Hoxa5 were associated with inflammation and thermogenesis, and especially TNFα, TLR4, and NLRP3 were significantly reduced, as well as adipose browning marker genes UCP1, PGC1α, and Dio2 were increased (Figure 1A). The subsequent GO analysis and pathway enhancement analysis showed that the distinct difference genes were enriched in encoding factors involved in TNF signal (Figure 1B) and TLR signal (Figure 1C). We further measured the mRNA expression of top affected genes in the RNA-seq samples by qPCR. The results showed that inflammation indicators IL1β, IL6, TNFα, TLR4, NF-κB, and NLRP3 were reduced by Hoxa5 (Figure 1D). Also the thermogenic-related genes UCP1, PGC1α, and Dio2 were enhanced by Hoxa5 (Figure 1E). Interestingly, TNC, which is an activator of TLR4, was also decreased by Hoxa5. These findings indicated that Hoxa5 is involved in both immune response and browning in adipocytes.

### Hoxa5 Promotes WAT Browning and Inhibits WAT Inflammation in Mice

**Figure 2:**
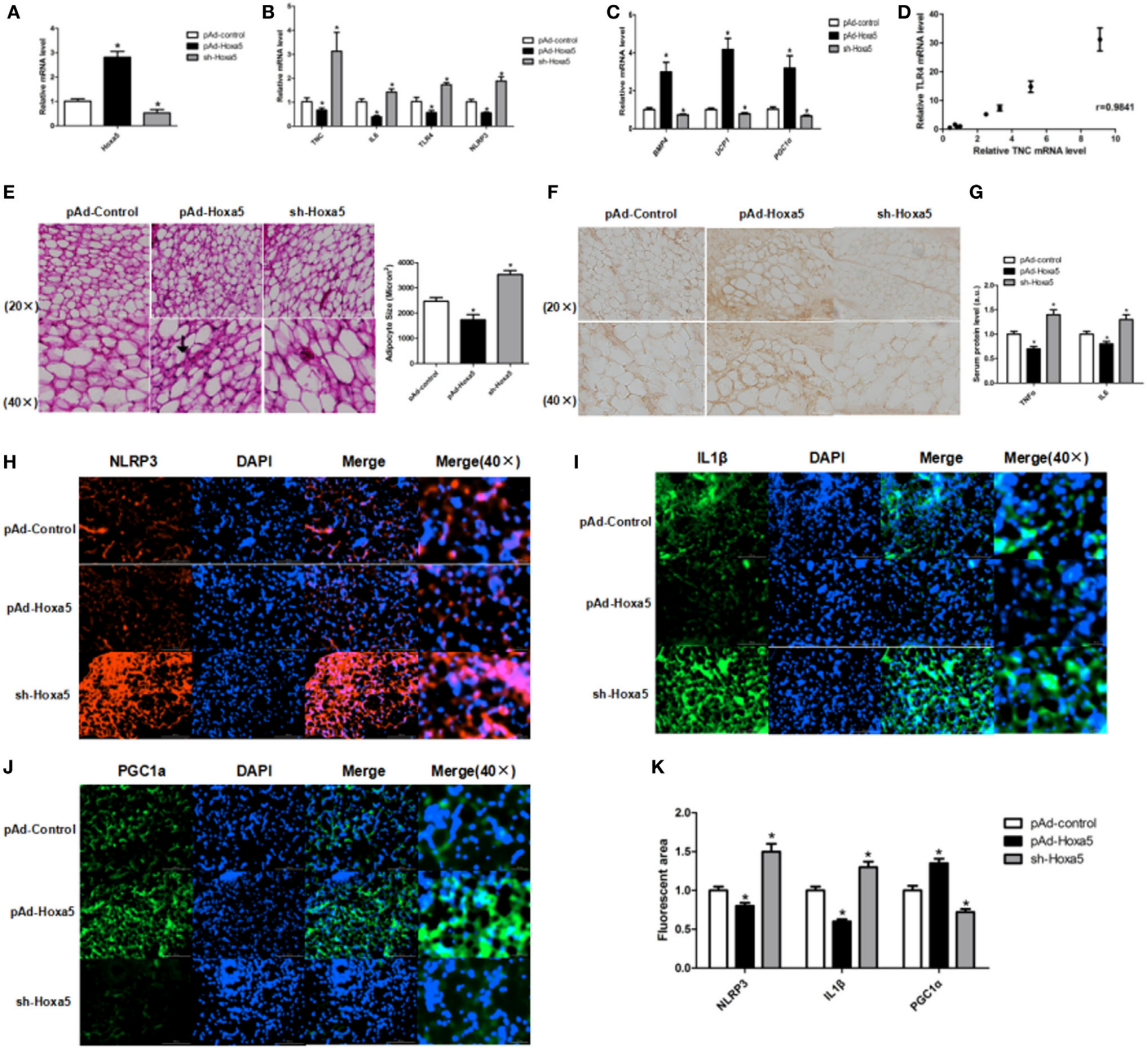
Homeobox a5 (Hoxa5) promotes white adipose tissue (WAT) browning and inhibits WAT inflammation in mice. All mice were injected with lipopolysaccharide and cold exposure. **(A)** Hoxa5 mRNA level of inguinal white adipose tissue (iWAT) in mice (*n* = 8). **(B)** Gene mRNA expression of TNFα, IL6, Toll-like receptor (TLR) 4, and NLRP3 in iWAT (*n* = 8). **(C)** Relative mRNA level of BMP4, UCP1, and PGC1α in mice iWAT in different groups (*n* = 8). **(D)** Correlation analysis of TLR4 and tenascin C (TNC) mRNA level in mice iWAT (*n* = 10). **(E)** Adipocyte size was monitored in frozen-section iWAT samples by hematoxylin-eosin staining (*n* = 8). **(F)** Images of immunohistochemical staining of iWAT in different groups. **(G)** Serum levels of TNFα and IL6 in different groups (*n* = 6). **(H–J)** Images of iWAT stained by immunofluorescent staining in different groups (*n* = 4). **(K)** Quantitate of immunofluorescent staining in Figures 3H–G. Values are represented as means ± SD vs. control group, *p < 0.05.

To further study the effects of Hoxa5 on inflammation and WAT browning, pAd-Hoxa5 injection with both LPS injection and cold exposure were performed on mice. Injection of pAd-Hoxa5 reduced mRNA levels of TNC, IL6, TLR4, and NLRP3 (Figures 2A,B; *p* < 0.05). At the same time, overexpression of Hoxa5 increased mRNA levels of BMP4, UCP1, and PGC1α (Figure 2C; *p* < 0.05), suggesting the activation of adipose browning with suppression of inflammation. In addition, the result showed mRNA levels of TNC and TLR4 in WAT were positively correlated, and the correlation coefficient is 0.9814 (Figure 2D). By H&E staining, we found that the adipocytes in iWAT are smaller in pAd-Hoxa5 injection group than that in control group (Figure 2E). Notably, immunofluorescence analysis of PGC1α in iWAT demonstrated that higher PGC1α protein level in the pAd-Hoxa5 group (Figure 2F). The serum protein levels of both TNFα and IL6 were reduced by Hoxa5 (Figure 2G). Moreover, the immunohistochemical analysis of iWAT revealed obvious decreases of NLRP3 and IL1β, and a strong increase of PGC1α staining by pAd-Hoxa5 injection (Figures 2H–K). These data revealed that Hoxa5 inhibits inflammation and promotes adipose browning in mice adipose tissue.

### Hoxa5 Enhances WAT Browning by Alleviating LPS-Induced Inflammation in Mice

**Figure 3:**
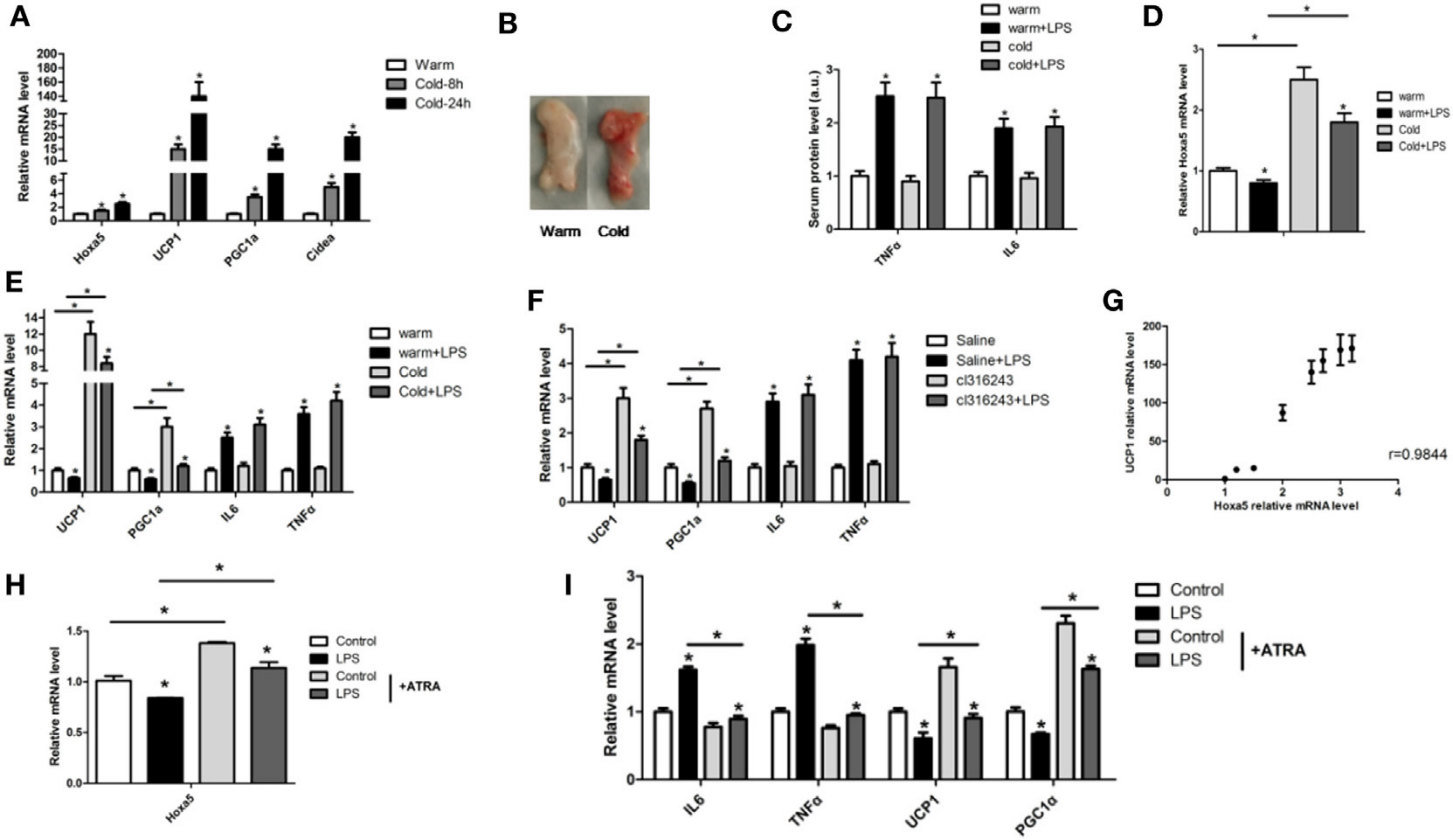
Homeobox a5 (Hoxa5) enhances white adipose tissue (WAT) browning by alleviating lipopolysaccharide (LPS)-induced inflammation in mice. **(A)** Relative mRNA levels of Hoxa5, UCP1, PGC1α, and Cidea in inguinal white adipose tissue (iWAT) after 8 or 24 h cold exposure at 4°C (*n* = 6). **(B)** iWAT representative picture of male mice with cold exposure at 4°C or 25°C for control (*n* = 6). **(C)** Mice with an injection of LPS at 4°C or 25°C, relative mRNA levels of Hoxa5 in iWAT of mice in different groups (*n* = 6). **(D)** Serum protein levels of TNFα and IL6 in different groups (*n* = 6). **(E)** UCP1, PGC1α, IL6, and TNFα mRNA expression in iWAT of mice injected with or without LPS at 4°C or 25°C (*n* = 6). **(F)** mRNA expressions of UCP1, PGC1α, IL6, and TNFα in iWAT of mice injected with or without LPS under cl316,243 treatment (*n* = 6). **(G)** Correlation analysis of UCP1 and Hoxa5 mRNA level in mice WAT (*n* = 6). **(H)** Hoxa5 relative mRNA expression in adipocytes with co-treatment of LPS and ATRA (*n* = 4). **(I)** Relative mRNA expressions of IL6, TNFα, UCP1, and PGC1α in adipocytes with co-treatment of LPS and ATRA (*n* = 4). Values are represented as means ± SD vs. control group, *p < 0.05.

To elucidate the effect of LPS-induced inflammation on adipose tissue browning, we first performed cold stimulation experiment, and the mice in the cold group were exposed to 4°C for 8 h or 24 h. Cold exposure increased the mRNA levels of Hoxa5, UCP1, PGC1α, and Cidea in iWAT (Figure 3A; *p* < 0.05). Tissue histology observation revealed that inguinal white tissue was smaller and darker in the cold group (Figure 3B). We established adipose tissue inflammation model by LPS with or without cold stimulation. The results showed that LPS increased both serum protein levels and mRNA levels of IL-6 and TNFα, while decreased mRNA levels of UCP1 and PGC1α in WAT (Figures 3C,E; *p* < 0.05). Furthermore, LPS reduced Hoxa5 mRNA level in iWAT (Figure 3D; *p* < 0.05). We further established another browning model by Cl316,243 injection, which showed consistent results (Figure 3F). By correlation analysis, the result showed that mRNA levels of Hoxa5 and UCP1 in WAT were positive correlated, and the correlation coefficient is 0.9844 (Figure 3G). We further performed Hoxa5 agonist-ATRA with LPS in adipocytes. Similarly, mRNA expressions of Hoxa5 and thermogenic markers UCP1 and PGC1α were downregulated accompanied by increased inflammation, and Hoxa5 agonist-ATRA reduced inflammation (Figures 3H,I; *p* < 0.05). The data indicated that LPS-induced inflammation reduced white adipose thermogenesis, and Hoxa5 enhanced WAT browning by alleviating LPS-induced inflammation.

### Hoxa5 Reverses the Inhibition of Adipocytes Browning in LPS Treated Adipocytes

**Figure 4:**
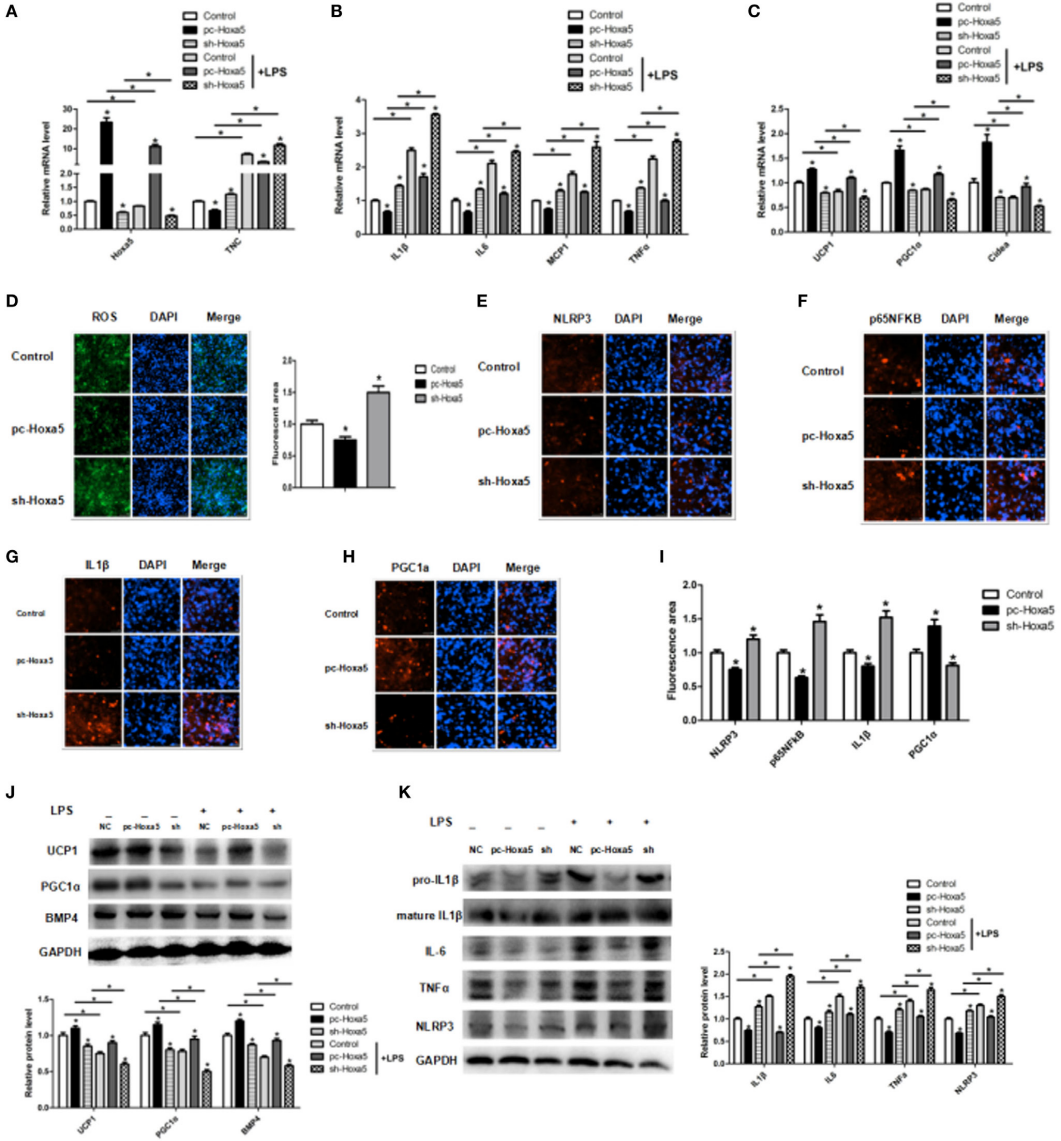
Homeobox a5 (Hoxa5) reverses the inhibition of adipocytes browning in lipopolysaccharide (LPS)-treated adipocytes. **(A–C)** mRNA expressions of Hoxa5, tenascin C (TNC), IL1β, IL6, MCP1, TNFα and UCP1, PGC1α, and Cidea in mice adipocytes in the pc-control group, pc-Hoxa5 group, and sh-Hoxa5 group with or without LPS treatment (*n* = 4). **(D)** Fluorescence staining of ROS in different groups with LPS treatment (*n* = 4). **(E–I)** Immunofluorescent staining of adipocytes in different groups with LPS treatment (*n* = 4). **(J)** Immunoblots images of UCP1, PGC1α, and BMP4 in different groups with LPS treatment (*n* = 4). **(K)** Immunoblots images of IL1β, IL6, TNFα, and NLRP3 in different groups with LPS treatment (*n* = 4). Values are represented as means ± SD vs. control group, *p < 0.05.

We further revealed the effect of Hoxa5 on adipocytes inflammation and browning *in vitro*. We detected the Hoxa5 and inflammatory factors expression after overexpressing Hoxa5 with LPS. LPS treatment decreased Hoxa5 mRNA level along with increasing TNC expression, and forced expression of Hoxa5 inhibited TNC mRNA level (Figure 4A; *p* < 0.05); co-processing of LPS and Hoxa5 inhibited the enhancement of IL1β, IL-6, MCP1, and TNFα (Figure 4B; *p* < 0.05) and elevated the reduction of UCP1, PGC1α, and Cidea (Figure 4C; *p* < 0.05), suggesting Hoxa5 had an inhibition effect on inflammation and a promotion role on browning. The reduction of ROS by Hoxa5 suggested the decreasing of inflammasome (Figure 4D; *p* < 0.05). Immunofluorescent staining showed that overexpression Hoxa5 markedly decreased protein expression of inflammation factors NLRP3, NF-κB, and IL1β and increased PGC1α protein expression (Figures 4E–I; *p* < 0.05). Consistently, we found that forced expression of Hoxa5 reduced protein levels of IL1β, IL6, TNFα, and NLRP3 (Figure 4K; *p* < 0.05) and increased protein levels of UCP1, PGC1α, and BMP4 (Figure 4J; *p* < 0.05). Thus, our results have suggested that Hoxa5 reversed the inhibition of adipocytes browning induced by LPS in adipocytes.

### Methylation Level of Hoxa5 Is Decreased Along With Increasing Browning in Adipose of Cold-Exposed Mice

**Figure 5:**
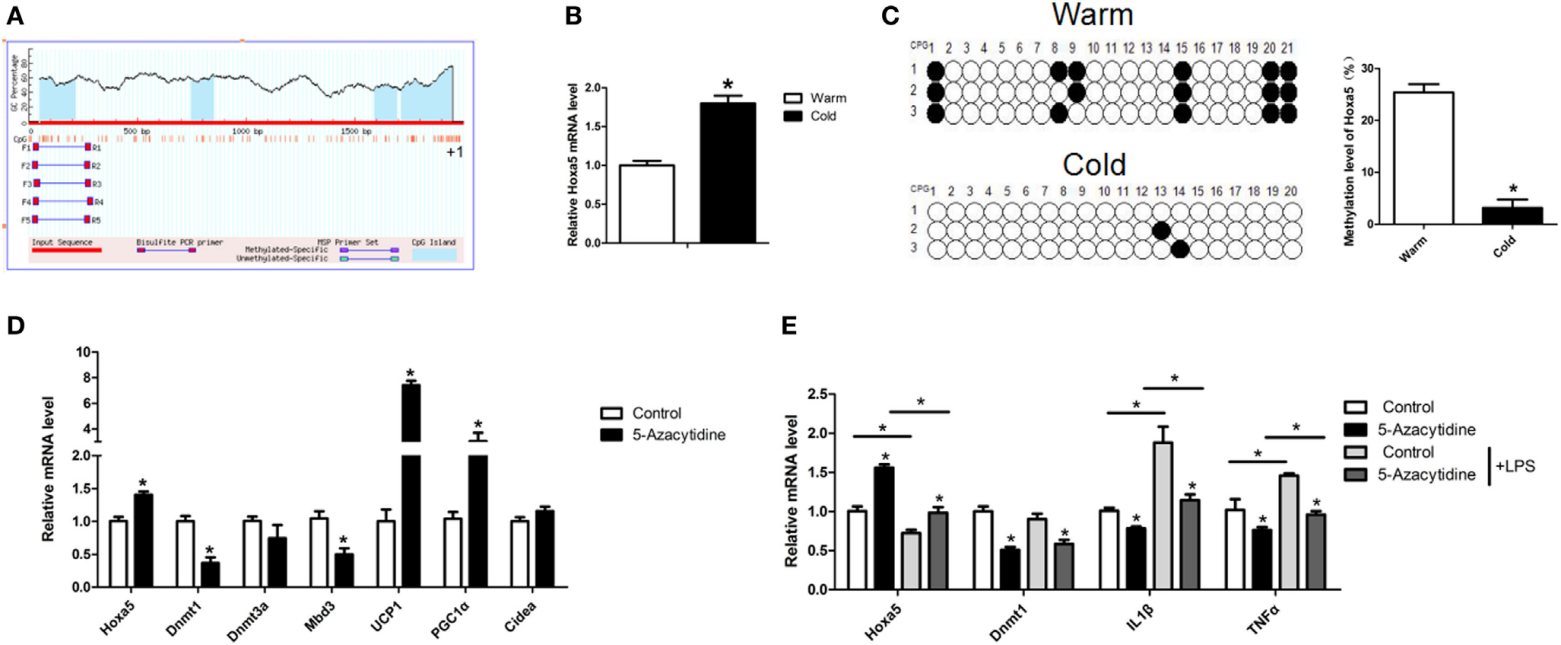
Methylation level of homeobox a5 (Hoxa5) is decreased along with increasing browning in adipose of cold exposed mice. **(A)** The schematic diagram of CpGs Hoxa5 promoter. **(B)** Relative mRNA level of Hoxa5 in inguinal white adipose tissue (iWAT) after 8 h cold exposure at 4°C (*n* = 6). **(C)** The methylation level of CpG in −148 bp ~ −14 bp of the Hoxa5 promoter in iWAT of mice with or without 8 h cold exposure at 4°C (*n* = 10). **(D)** Relative mRNA level of Hoxa5, Dnmt1, Dnmt3a, Mbd3, UCP1, PGC1α, and Cidea in iWAT with 5-AZA injection (*n* = 8). **(E)** Relative mRNA level of Hoxa5, Dnmt1, IL1β, and TNFα in adipocytes with or without 5-AZA under lipopolysaccharide (LPS) treatment (*n* = 4). Values are represented as means ± SD vs. control group, *p < 0.05.

Having found cold stimulation increased Hoxa5 expression (Figures 3A and 5B). To further study the effect of cold stimulation on methylation level of the Hoxa5 promoter, we studied methylation level of the Hoxa5 promoter (Figure 5A). The results showed that the methylation level of CpG in Hoxa5 promoter in cold-exposed iWAT is lower than that present in warm (Figure 5C; *p* < 0.05). Then, by injecting mice with methylation inhibitor—5-azacytidine—we found that mRNA levels of browning key genes UCP1 and PGC1α, as well as Hoxa5, were increased in iWAT; and mRNA levels of Dnmt1 and Mbd3 were inhibited (Figure 5D; *p* < 0.05). This result suggested the descend of Dnmt1 and Mbd3 elevated browning and Hoxa5 expression. We further treated adipocytes with 5-azacytidine and LPS, the results showed inflammation factors IL1β and TNFα were blocked by 5-azacytidine, which indicated the anti-inflammatory effect of 5-azacytidine on adipocytes (Figure 5E; *p* < 0.05). These data confirmed that the Hoxa5 expression increased in mice adipose tissue by descended methylation level in cold exposure.

### Hoxa5 Represses Adipocytes Inflammation by Negative Transcription Regulation of TNC

**Figure 6:**
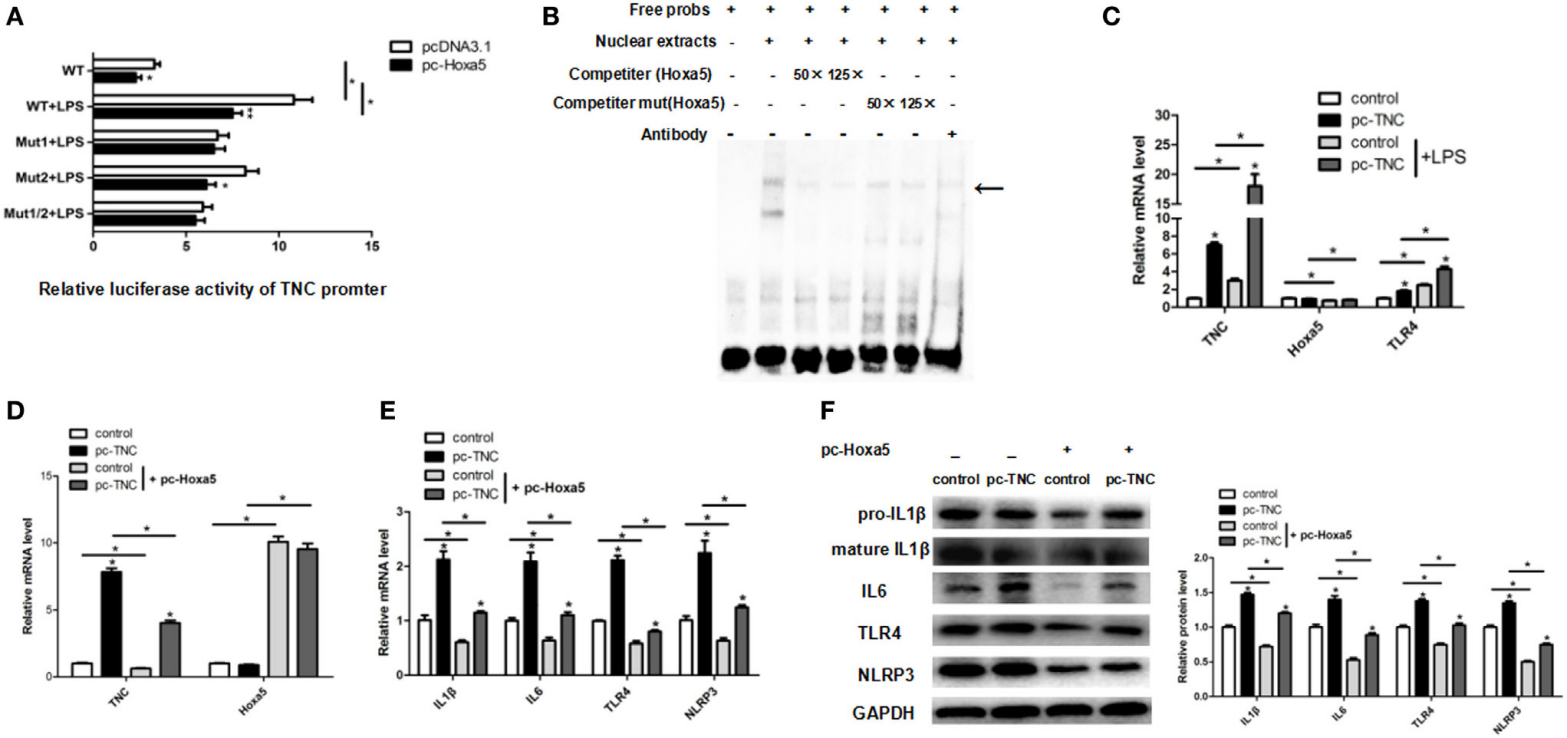
Homeobox a5 (Hoxa5) represses adipocytes inflammation by negative transcription regulation of tenascin C (TNC). **(A)** Mutational fragments of TNC promoter in a luciferase reporter vector in pc-control or pc-Hoxa5 groups (*n* = 4). **(B)** Electrophoretic mobility shift assay showing direct binding of Hoxa5 to TNC promoter *in vitro*. The main complexes are marked with arrows. **(C)** Relative mRNA expression level of TNC, Hoxa5, and Toll-like receptor (TLR) 4 in mice adipocytes in pc-control, pc-Hoxa5, and sh-Hoxa5 group with or without lipopolysaccharide (LPS) treatment (*n* = 4). **(D,E)** Relative mRNA expression level of TNC, Hoxa5, IL1β, IL6, TLR4, and NLRP3 in mice adipocytes co-treatment with pc-TNC and pc-Hoxa5 under LPS treatment (*n* = 4). **(F)** immunoblots images for IL1β, IL6, TNFα, and NLRP3 in mice adipocytes co-treatment with pc-TNC and pc-Hoxa5 under LPS treatment (*n* = 4). Values are represented as means ± SD vs. control group, *p < 0.05.

To explore the relationship between Hoxa5 and TNC, we forecasted potential transcription factors of TNC by Genomatix software. There were two potential binding sites (−477 bp and −228 bp) of Hoxa5 on the TNC promoter. The result of luciferase reporter assay showed that the 477 bp~460 bp upstream of TNC transcription start site may be the binding site of Hoxa5 (Figure 6A; *p* < 0.05). We further performed EMSA, and the result confirmed that Hoxa5 may combine on 477 bp~460 bp upstream of TNC transcription initiation site (Figure 6B). Overexpression of TNC enhanced TLR4 expression (Figure 6C; *p* < 0.05). After co-treatment of pc-TNC and pc-Hoxa5, the results showed forced expression of TNC elevated mRNA levels of inflammation factors IL1β, IL6, TLR4, and NLRP3, which were reversed by overexpression of Hoxa5 (Figures 6D,E; *p* < 0.05). Consistently, protein levels of IL1β, IL6, TLR4, and NLRP3 were increased by TNC, while inhibited by Hoxa5 (Figure 6F; *p* < 0.05). These data suggested that Hoxa5 represses adipocytes inflammation by negative transcription regulation of TNC.

### TNC Facilitates Adipocytes Inflammation *via* Activating the TLR4/NF-κB/NLRP3 Signal Pathway

**Figure 7:**
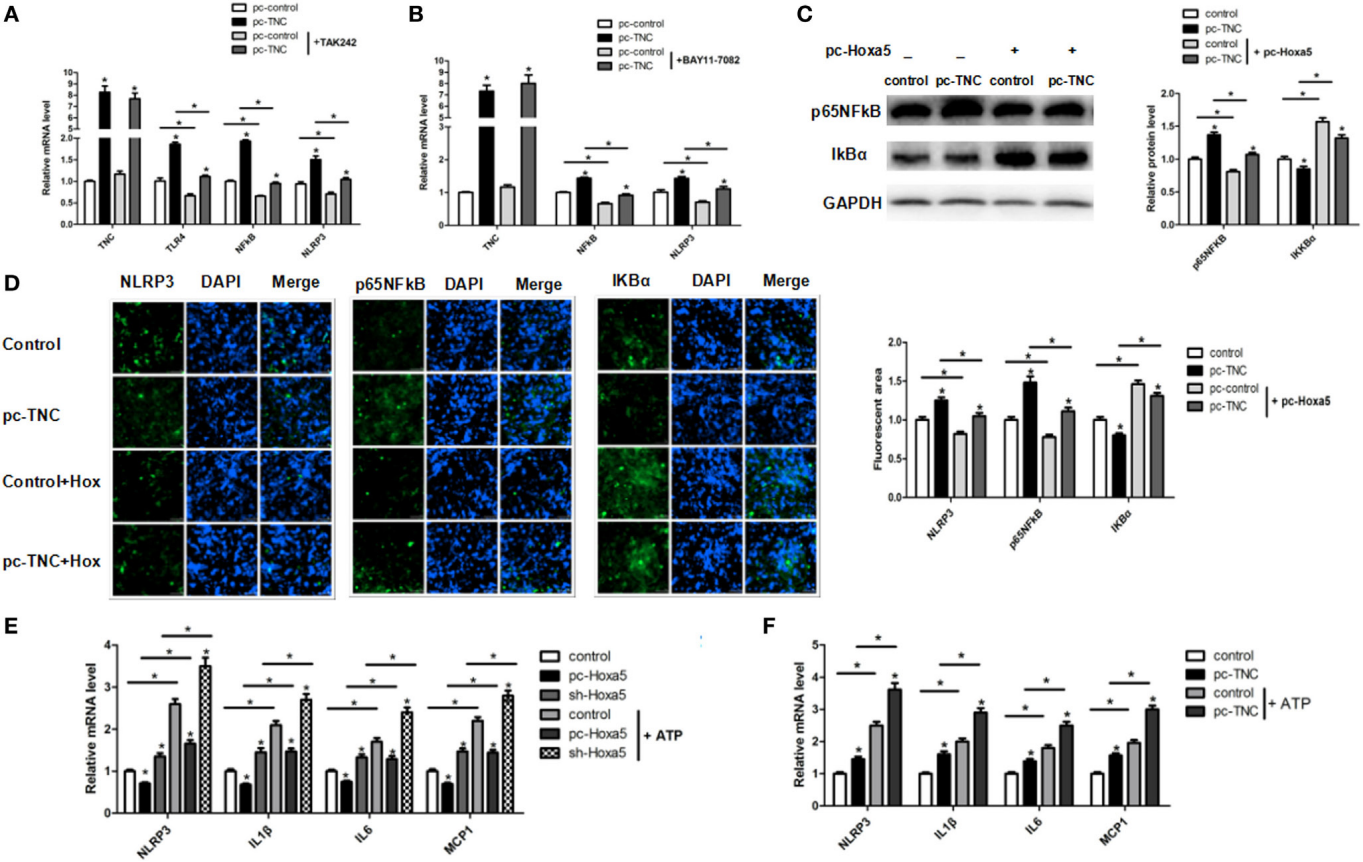
Tenascin C (TNC) facilitates adipocytes inflammation *via* activating the Toll-like receptor (TLR) 4/nuclear factor kappa B (NF-κB)/NLRP3 pathway. **(A)** Relative mRNA expression level of TNC, TLR4, and NF-κB in mice adipocytes in control group, a pc-TNC group with or without treatment of TLR4 inhibitor TAK242 (*n* = 4). **(B)** Relative mRNA expression level of TNC, NF-κB, and NLRP3 in mice adipocytes in control group, a pc-TNC group with or without treatment of NF-κB inhibitor BAY11-7082 (*n* = 4). **(C)** Representative immunoblots and densitometric quantification for IL1β, IL6, TNFα, and NLRP3 in mice adipocytes co-treatment with pc-TNC and pc-Hoxa5 under lipopolysaccharide (LPS) treatment (*n* = 4). **(D)** Images of adipocytes stained by immunofluorescent staining in mice adipocytes co-treatment with pc-TNC and pc-Hoxa5 under LPS treatment (*n* = 4). **(E,F)** Relative mRNA level of NLRP3, IL1β, IL6, MCP1 in mice adipocytes in different groups with or without ATP treatment under LPS stimulation (*n* = 4). Values are represented as means ± SD vs. control group, *p < 0.05.

We then studied the effect of TNC on TLR4/NF-κB signal pathway with the TLR4-specific inhibitor TAK-242 and NF-κB-specific inhibitor BAY11-7082, respectively. The mRNA level of both TLR4 and NF-κB were increased by overexpression of TNC, regardless of the presence of TAK-242 treatment (Figure 7A; *p* < 0.05). Moreover, TNC enhanced the mRNA level of NF-κB and NLRP3, regardless of the presence of BAY11-7082 treatment; BAY11-7082 treatment also decreased NF-κB and NLRP3 mRNA expression (Figure 7B; *p* < 0.05). After co-treatment of pc-TNC and pc-Hoxa5, the results showed that forced expression of TNC activated NF-κB and blocked IĸBa, which were reversed by overexpression of Hoxa5 (Figure 7C; *p* < 0.05). Consistently, immunofluorescent staining showed that overexpression of TNC markedly increased protein expression of inflammation factors NLRP3 and NF-κB and reduced IĸBa, which were reversed by overexpression of Hoxa5 (Figure 7D; *p* < 0.05). We further detected the mRNA level of LPS-induced inflammation factors under inflammasome agonist-ATP. The results showed that the activation of NLRP3 inflammasome strongly enhanced inflammation, and Hoxa5 inhibited the expression of NLRP3 and inflammation factors IL1β, IL6, and MCP1, regardless of the presence of ATP (Figure 7E; *p* < 0.05). TNC increased the mRNA level of NLRP3, IL1β, IL6, and MCP1, no matter whether with ATP or not (Figure 7F; *p* < 0.05). These results implied that TNC facilitates adipocytes inflammation *via* activating the TLR4/NF-κB/NLRP3 signal pathway.

### Hoxa5 Promotes Adipocytes Browning by Activating the BMP4/Smad1 Signal Pathway

**Figure 8:**
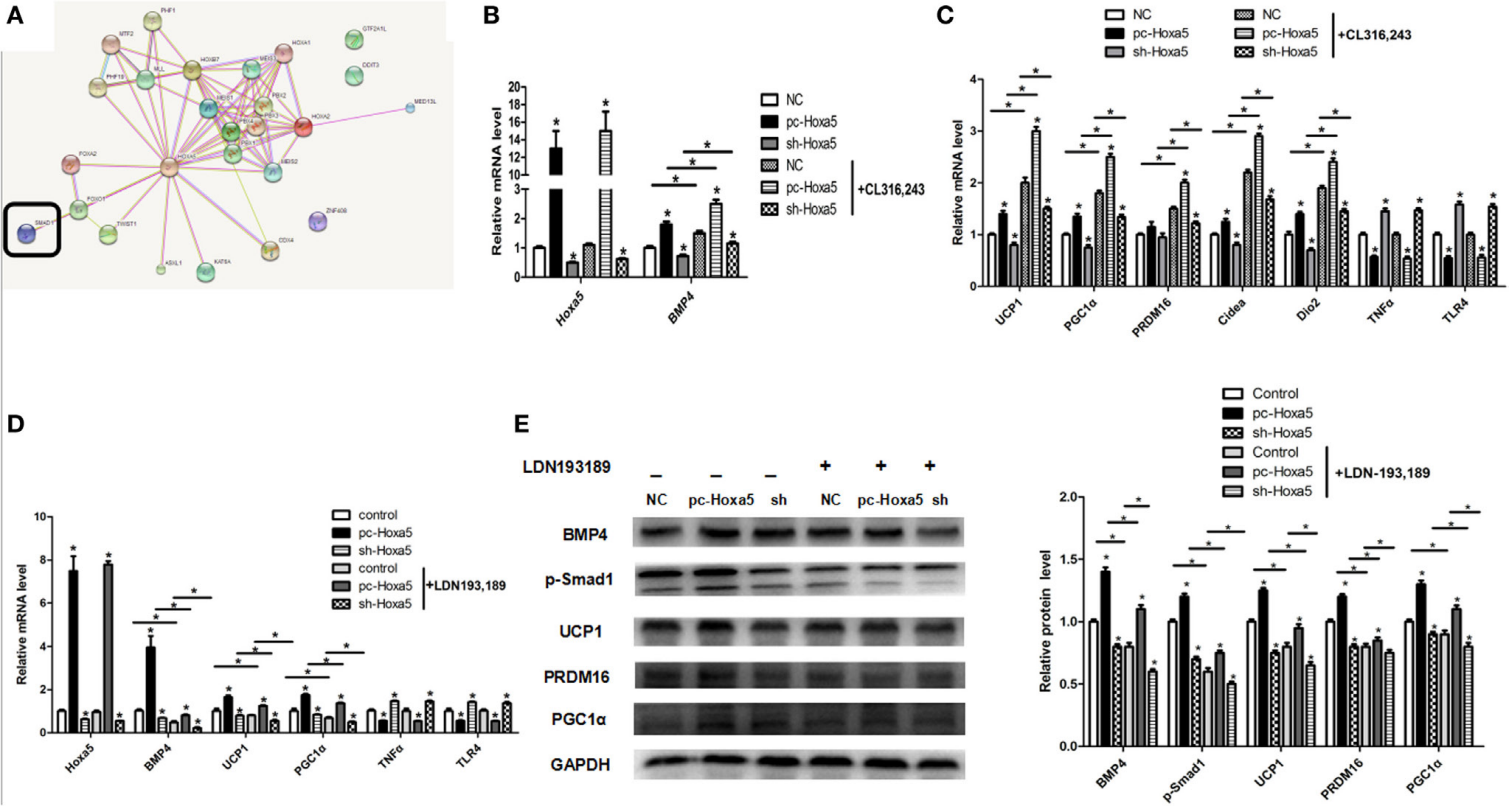
Homeobox a5 (Hoxa5) promotes adipocytes browning by activating the BMP4/Smad1 signal pathway. **(A)** The network of the target genes of Hoxa5 by bioinformatics analysis. **(B,C)** mRNA expressions of Hoxa5, BMP4, UCP1, PGC1α, PRDM16, Cidea, Dio2, TNFα, and Toll-like receptor (TLR) 4 in mice adipocytes in the pc-control group, pc-Hoxa5 group, and sh-Hoxa5 group with or without β3-adrenoceptor agonists CL314,243 (*n* = 4). **(D)** Relative mRNA level of Hoxa5, BMP4, UCP1, PGC1α, TNFα, and TLR4 in mice adipocytes in control group, pc-Hoxa5 group, and sh-Hoxa5 group with or without Smad1 inhibitor LDN193,189 (*n* = 4). **(E)** Representative immunoblots and densitometric quantification for BMP4, p-Smad1, UCP1, PRDM16, and PGC1α in mice adipocytes in pc-control group, pc-Hoxa5 group, and sh-Hoxa5 group with or without DN193,189 (*n* = 4). Values are represented as means ± SD vs. control group, *p < 0.05.

To further understand how Hoxa5 increases adipocytes browning, we established a browning model by β3-adrenoceptor agonist CL316,243 *in vitro* study. Not surprisingly, overexpression of Hoxa5 enhanced mRNA levels of browning key factors BMP4, UCP1, PGC1α, PRDM16, Cidea, and Dio2, regardless of the presence of CL316,243 treatment (Figures 8B,C; *p* < 0.05). The expression of increased thermogenic factors induced by Hoxa5 prompted us to assume that there is another pathway in the adjustment of adipose tissue browning. From the bioinformatics analysis, we found that Hoxa5 interacted with Smad1 (Figure 8A). To explore whether Smad1 is account for Hoxa5-mediated adipocytes browning, we then performed forced expression of Hoxa5 along with Smad1 signal inhibitor—LDN193,189. The treatment of LDN193,189 markedly reduced mRNA level of BMP4, UCP1, and PGC1α (Figure 8D; *p* < 0.05). Importantly, to confirm that the effect of Hoxa5 on inflammation is not through WAT browning, we also detected the effect of Hoxa5 on inflammation with the treatment of CL316,243 or LDN193,189. As expected, mRNA levels of TNFα and TLR4 were not altered by CL316,243 or LDN193,189 (Figures 8C,D). The protein levels of BMP4 and phosphorylation of Smad1, as well as browning markers UCP1, PGC1α, PRDM16, were increased by Hoxa5, regardless of the presence of LDN193,189; At the same time, LDN193,189 reduced phosphorylation level of Smad1 as well as protein expression of BMP4, UCP1, PGC1α, and PRDM16 (Figure 8E; *p* < 0.05). Overall, these findings implicated that Hoxa5 promotes adipocytes browning by activating the BMP4/Smad1 signal pathway.

## Discussion

Lipopolysaccharide induces inflammatory reactions and increases many cytokines and chemokines *via* TLRs (27). In this study, we found that TLRs were significantly decreased by Hoxa5, and then we performed LPS-induced inflammation model to explore the molecular processes affected by Hoxa5 during inflammation. WAT or beige adipocytes browning burn fat and dissipate the energy as heat, which is in favor of loss weight as well as glucose homeostasis amelioration (28, 29). Here, we found LPS-induced inflammation and damaged WAT browning in mice, which is in line with previous reports (30–32). Moreover, we demonstrated that Hoxa5 inhibited LPS-induced inflammation in WAT. Then we have proved that Hoxa5 takes part in the regulation of inflammasome and enhances WAT browning. The research declared an important relationship between Hoxa5 and inflammation or browning in adipose tissue, which has not been studied.

Homeobox a5 is a vital transcription factor, which is highly expressed in the adipose tissue, and plays an important part in regulating adipocytes, including differentiation and adipose distribution (33, 34). Herein, we found that Hoxa5 decreased mRNA levels of TNC and inflammation factors by RNA-seq analysis. TNC, a kind of extracellular matrix glycoprotein, specifically expresses at a high level in acute inflammation (35, 36). TLRs stand for a crucial molecular relationship between LPS stimulation and inflammation (37). Importantly, TNC displays pro-inflammatory effects mediated by the activation of TLR4 (38). In this study, we demonstrated that Hoxa5 has a strong inhibition effect on TLR4 by transcriptional suppressing of TNC, leading a promoting result on adipose tissue browning.

Inflammasome, a group of protein complexes, functions as a sensor to detect danger signals and induces secretion of potent pro-inflammatory cytokines that contribute to obesity-associated chronic inflammation conditions (3, 39, 40). The NF-κB signal pathway which was correlated with inflammatory response and has been identified binding to NLRP3 promoter and transcriptional regulating NLRP3 and its downstream targets (41, 42). In this study, we determined that Hoxa5 may take part in the regulation of NLRP3 inflammasome by modulating the NF-κB signal in adipocytes.

Studies showed that overexpression of BMP4 in white adipocytes lead to reduction of WAT and cell size (43, 44). Here, our data indicated that Hoxa5 markedly activated BMP4-mediated Smad1 signal pathway, giving positive effect adipose browning. While the underlying relationship between Hoxa5 and BMP4 still need to be revealed.

The previous study has reported a reduction of Hoxa5 mRNA level in WAT along with the increased methylation level in Hoxa5 promoter after HFD in mice (45). While inhibiting DNA methylation by 5-Aza-2′-deoxycytidine suppressed macrophage inflammation (46). Interestingly, we found a novel link between decreased Hoxa5 promoter’s methylation level in WAT and cold exposure. In addition, we determined the promoting effect of methylation inhibitor 5-AZA on WAT browning as well as the inhibition of inflammation in adipocytes.

**Figure 9:**
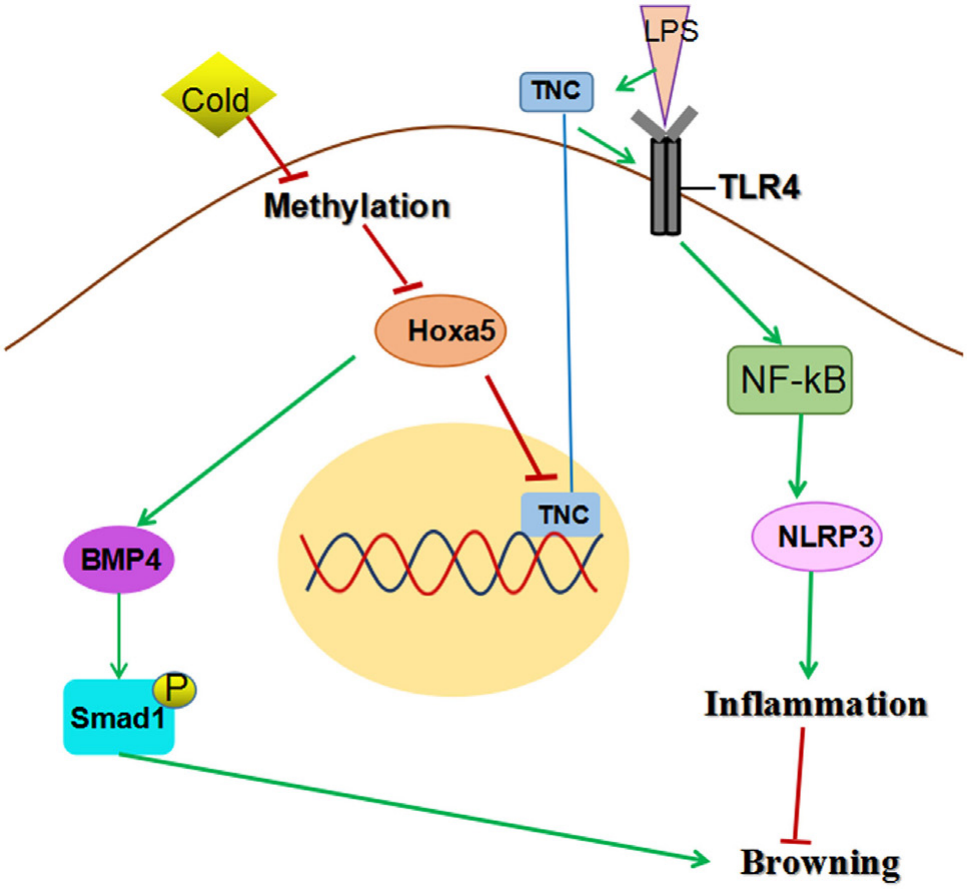
Homeobox a5 (Hoxa5) promotes white adipose tissue browning through reducing tenascin C (TNC)/toll-like receptor (TLR) 4/nuclear factor kappa B (NF-κB) inflammatory signaling in mice. Hoxa5 promotes adipose tissue browning by activating BMP4/Smad1 signal and alleviating inflammation *via* inhibiting TNC/TLR4/NF-κB pathway.

In conclusion, our results demonstrated that Hoxa5 promotes adipose tissue browning by inhibiting the TNC/TLR4/NF-κB inflammatory signaling in mice; in addition, we found that the Hoxa5 was a novel activator of the BMP4/Smad1 pathway (summarized in Figure 9). These findings may help to uncover a novel role of Hoxa5 in the regulation of adipose tissue.

## Data Accession

The raw data have been deposited to NCBI Sequence Read Archive (SRA). The NCBI SRA accession: SRP134917.

## Ethics Statement

Mice handling protocols were conducted following the guidelines and regulations approved by the Animal Ethics Committee of Northwest A&F University.

## Author Contributions

WC: design, data collection, analysis, and writing manuscript; HH and TX: data collection and analysis; CL: analysis; SM: writing manuscript; CS: design.

## Conflict of Interest Statement

The authors declare that the research was conducted in the absence of any commercial or financial relationships that could be construed as a potential conflict of interest.
